# Anomalous bond length behavior and a new solid phase of bromine under pressure

**DOI:** 10.1038/srep25649

**Published:** 2016-05-09

**Authors:** Min Wu, John S. Tse, Yuanming Pan

**Affiliations:** 1 College of Materials Science and Engineering, Zhejiang University of Technology, Hangzhou, 310014, P. R. China; 2 Department of Physics and Engineering Physics, University of Saskatchewan, Saskatoon, Saskatchewan S7N 5E2 Canada; 3 Department of Geological Sciences, University of Saskatchewan, Saskatoon, Saskatchewan S7N 5E2 Canada; 4 State Key Laboratory of Superhard Materials, Jilin University, Changchun 130012, P. R. China

## Abstract

The behavior of diatomic molecular solids under pressure have attracted great interest and been extensively studied. Under ambient pressure, the structure of bromine is known to be a molecular phase (phase I). With increasing pressure, it transforms into an incommensurate phase (phase V) before eventually to a monoatomic phase (phase II). However, between phases I and V, the interatomic distance was found to first increase with pressure and then decreased abruptly. This anomalous bond length behavior is accompanied by the splitting of the Raman bands. These phenomena have not been resolved. Here we suggest a new solid phase that explains the Raman spectra. Furthermore, the anomalous bond length behavior is found to be the result of subtle second neighbor intermolecular interactions and is an intrinsic property of bromine in molecular phases.

The behaviors of diatomic molecular solids under pressure have attracted great interests^1^,^2^,^3^,^4^,^5^,^6^,^7^. Molecular solid hydrogen was expected to be metallized and dissociate into an atomic phase at extremely high pressure (~0.4 TPa)^1^,^2^, although it has not yet been proven by experiments. For heavier halogens (Br_2_ and I_2_), the known sequence of phase transitions is from the ambient molecular solid (phase I) to an incommensurate phase (phase V) and is eventually dissociated into a monoatomic phase (phase II). From the Raman spectra^5^, vibrational bands belonging to a new structural phase were found preceding the onset of the transformation to phase V at 12.5 GPa for iodine and 25 GPa for bromine. Based on first-principles calculations and the re-investigation of the X-ray diffraction (XRD) pattern of iodine^7^, a new solid phase *C*2/*m* was proposed in which the calculated vibrational frequencies matched the observed Raman splitting. On the other hand, the origin of a similar splitting in the Raman peaks in bromine at ~25 GPa remains unclear^5^. This feature was speculated to be associated with a weakly modulated structure as changes in the electronic properties were reported to occur at a similar pressure from Br K-edge X-ray absorption spectra^8^. However, this suggestion contradicts the results from X-ray diffraction study, in which no noticeable structural change was found^9^. On the other hand, analysis of the extended X-ray absorption fine structure at 25 GPa hinted that a phase transition may have occurred^6^. The most interesting observation was that the Br-Br bond length was found to increase with pressure from ambient condition up to ~25 GPa. Incidentally, a similar increase in the bond length has also been reported for I_2_^10^. The increase of intramolecular bond length with pressure generally is correlated to the increase of intermolecular interaction and this would lead to the eventual molecule dissociation and formation of the monoatomic phase. An interesting and surprising aspect in Br_2_ is that the Br-Br bond length starts to decrease with further compression before the onset of dissociation into the monoatomic phase. A Raman study had revealed that, at 25 GPa several vibrational bands had split into two or more peaks, suggesting a structural phase transition^5^. Previous theoretical calculations seems to reproduce the trend in the bond length variation, however, no analysis were performed to understand this anomalous behavior^11^,^12^.

A reduced volume model has been proposed to explain the Raman spectra of compressed solid bromine and iodine^6^. However, this simple model only established an empirical correlation but did not explain the trend in the variation of the intramolecular bond length. A detailed theoretical investigation is needed to clarify the exact nature of the bond length reversal and the splitting of the Raman bands. In the present study, first-principles calculations have been performed to investigate the structures and electronic properties of solid bromine under pressure. The unusual bond length variation is reproduced and shown to be related to the arrangement of bromine molecules where the second neighbor intermolecular interaction becomes more important with increasing pressure. Furthermore, the observed splitting in the A_g_^(L)^ and B_3g_^(L)^ Raman bands is shown to be unrelated to the bond length change. Instead, it is due to a structural transformation from the *Cmca* to a *C*2/*m* structure, similar to that proposed for solid iodine. Our calculations also found that the metallization of bromine should occur at a much higher pressure than that observed for the anomalous reversal of the bond length and has no direct relationship with the latter as hypothesized earlier.

## Results and Discussion

### Equation of states

**Figure 1:**
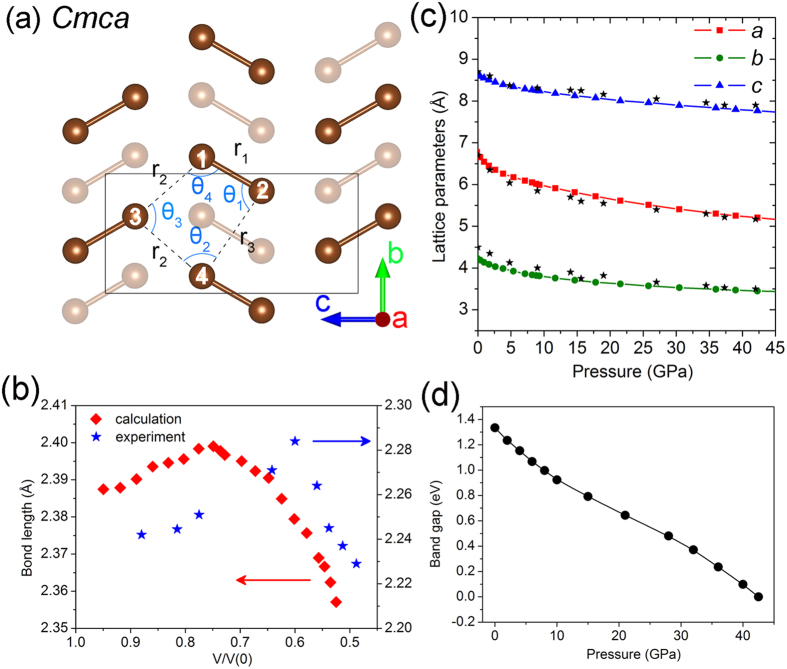
(**a**) The *Cmca* structure of Br_2_. The light colored atoms are in the second layer. r_1_, r_2_ and r_3_ represent the intramolecular bond length, first neighbor intermolecular distance and second neighbor intermolecular distance, respectively. (**b**) Bond length evolution comparison between calculated result from PBE-D2 method (red square) and experiment result (blue star). V is the cell volume and the reference V(0) is the volume at ambient pressure in experiment (see text). (**c**) The calculated and experimental lattice parameters evolution under pressure. The colored dots and the black stars represent the calculated and experimental results, respectively. The experimental values are from ref.^9^.(**d**) Calculated pressure dependence of the band gap energy using PBE+ D2 method for *Cmca* bromine phase. The solid line is the guide for eye.

Figure 1a shows the ambient pressure *Cmca* molecular bromine structure. The unit cell contains 8 Br atoms and the crystal structure is constructed from repeated layers of bromine molecules arranged in zigzag chains parallel to the ***bc*** plane and stack along the crystallographic *a* axis. The equation of states (EOS) of the *Cmca* structure calculated from different exchange-correlation and van der Waals (vdW) functionals (see method section for details) are shown in Fig. S1a. No density functional can reproduce the experimental results exactly. Comparing the calculated and experimental EOS and structural parameters^9^ (Table S1, see supplementary information), it is found that the Grimme D2 correction (PBE+ D2) provided the most reliable results over the pressure range of interest. Similar results have been reported recently on the ground state of crystalline bromine^13^. Hereafter, results obtained using the PBE+ D2 method will be presented and discussed as this combination, although not perfect, it gave the best agreement with the experimental ambient unit cell parameters. The calculated volume of the *Cmca* unit cell at ambient pressure with the PBE+ D2 method of 245 Å^3^ is ~6% smaller than the observed value of 260 Å^3^. A smaller volume indicates that even with vdW correction the intermolecular interactions are overestimated. The calculated lattice parameters at ambient pressure *a* =  6.738 Å, *b* =  4.217 Å and *c* =  8.628 Å are comparable with the experimental values of *a* =  6.670 Å, *b* =  4.480 Å and *c* =  8.720 Å^9^ except the length of the predicted crystallographic ***b*** axes is noticeably shorter. Figure 1c compares the calculated and observed lattice parameters from ambient pressure to 45 GPa. The overall agreement is satisfactory, and again the predicted ***b*** axes deviated from the experiments noticeably, particularly at pressures less than 25 GPa. Thus, the densities of the calculated structures are denser than the experiments, and therefore the theoretical pressures were underestimated. In an alternate viewpoint, a smaller calculated ambient volume can be rationalized as the theoretical structure was already pre-compressed.

### Anomalous bond length evolution

**Figure 2:**
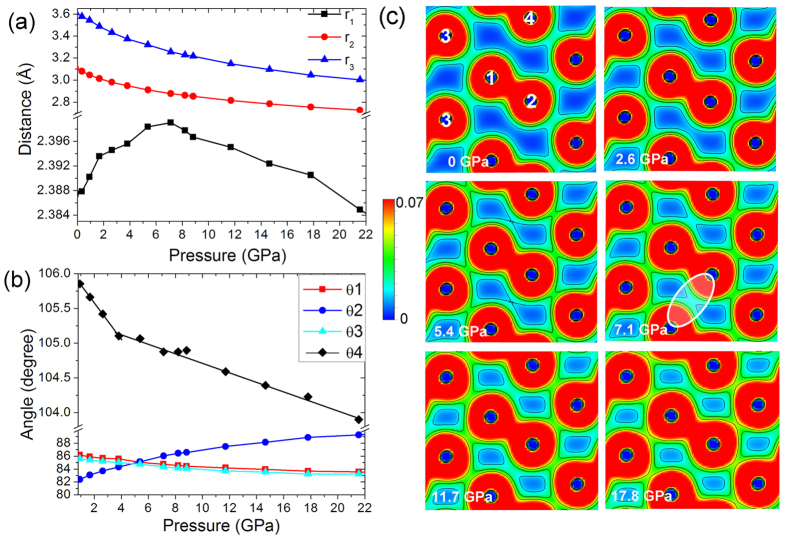
(**a**) Evolution of intermolecular distances under pressure in the Cmca structure. (**b**) Intermolecular angles under pressure. The labels for the distance and angle are illustrated in Fig. 1a. (**c**) 2D charge density plots of the bromine Cmca structure at selected pressures. The unit of the charge density is e/cell. The emergence of the second intermolecular interaction is highlighted with a white ellipse.

In spite of the pre-compression effect discussed above, it is significant that the anomalous evolution of the intramolecular Br_2_ (< Br-Br> ) bond lengths (*i.e*. an initial lengthening followed by shortening with increasing pressure) reported in EXAFS experiments^6^ is correctly reproduced (Fig. 1b). The calculated < Br-Br> bond length at ambient pressure of ~2.387 Å is longer by ~5% than the experimental value of ~2.27 Å due to the overestimated intermolecular interactions (*vide supra*). Consequently, the reversal of the average bond length (Fig. 2b) predicted at ~6 GPa is lower than the observed pressure of 25 GPa. Instead of using the absolute pressure scale, we choose to compare the bond length evolution under compression to the calculated unit cell volume reference to that at ambient pressure (*V*/*V*(0)). As shown in Fig. 1b, both theory and experiment show an initial increase of the Br-Br bond length and the trend is reversed when the volume is reduced to 60–75%. This anomalous bond length trend observed and predicted in the *Cmca* phase is not related to an insulator-metal transition as speculated earlier^6^. This is supported by the calculated band gap of the *Cmca* structure using PBE+ D2 method as a function of pressure (Fig. 1d) in which the metallization is estimated to be at ~42.5 GPa, much higher than the pressure when the Br-Br bond length trend was reversed. The comparisons and discussions presented above show, although the PBE+ D2 vdW correction is not perfect and requires a rescaling in pressure, the predicted structural changes are reliable for further investigation.

To understand the anomalous bromine bond length behavior, we have constructed several model calculations. A 1D model of bromine arranged in a head-to-tail fashion was studied. The evolution of the intramolecular distance with compression presented in Fig. S2 shows the intramolecular distance increases monotonically with pressure and then dissociates into a monoatomic chain. No decrease of the intramolecular distance is found before the onset of dissociation.

We then investigated the effect of the interlayer interaction on the Br_2_ bond length. For this purpose a single 2D layer of bromine extracted from the *Cmca* structure (Fig. 1a) is compressed. With this model, the anomalous bond length (*r*_1_) behavior is reproduced (Fig. S3). In this case, the intramolecular distance at ambient pressure is found to be further overestimated by 0.2% (*i.e*., 2.392 Å) compare to the 3D *Cmca* structure. Consequently, the calculated transition pressure of ~4 GPa is further reduced. The results show the interlayer interaction is not solely responsible for the anomalous bond length behavior.

### Charge density analysis

In the 3D *Cmca* structure, the first intermolecular neighbor (*r*_2_, between Br(1) and Br(3) and second intermolecular neighbor distances (*r*_3_, between Br(2) and Br(4)) depicted in Fig. 2a are found to decrease continuously with pressure. *r*_2_ decreased from the initial value of 3.108 Å at ambient with increasing pressure and this help to enhance intermolecular interactions and resulted in longer intramolecular Br-Br bond lengths. On the other hand, the second intermolecular neighbor *r*_3_ is 3.614 Å at ambient pressure and therefore the second neighbor interaction is much weaker. However, upon compression, *r*_3_ decreased faster than *r*_2_ and at the turnover of the intramolecular bond length, *r*_3_ decreased to 3.256 Å, which is comparable to *r*_2_ at ambient pressure. This observation shows the contribution of the second neighbor intermolecular interaction to the structural change will become more important at higher pressures. This is supported by charge density analysis of the *Cmca* structure at several pressures (Fig. 2c). The second neighbor intermolecular interaction between atoms 2 and 4 starts to emerge until the pressure increases up to 7.1 GPa as highlighted in Fig. 2c. The closer contact with the second nearest intermolecular neighbor changed the relative orientation of the bromine molecules upon compression. Fig. 2b shows significant changes in all the intermolecular angles (illustrated in Fig. 1a). In particular, the interatomic angle θ _4_ of Br(3)-Br(1)-Br(2) atoms decreased rapidly until *ca*. 5 GPa, incidentally, close to the theoretical pressure for the reversal of the Br_2_ bond length. The application of external pressure forced the bromine molecule (Br(1)-Br(2)) to rotate clockwise and led to the decrease of the second neighbor distance *r*_3_. When the pressure approaches the bond length reversal, the second neighbor interaction has increased significantly and hindered further rotation of the bromine molecules. It can be concluded that the increasing role of the second neighbor intermolecular interaction is the fundamental reason for the anomalous bond length trend.

### Raman spectra calculations

Figure S4 shows the calculated Raman spectra of the *Cmca* structure at selected pressures. At ambient pressure when the intermolecular interactions are very weak almost no active librational Raman mode is visible. As the pressure is increased, a low frequency librational mode A_g_^(L)^ starts to emerge which is followed by a second librational mode B_3g_^(L)^ at 5.4 GPa. The appearance of the latter mode is consistent with the calculated bond length reversal as the librational motion of B_3g_^(L)^ is influenced by the second neighbor intermolecular interaction. The hysteretic appearance of the B_3g_^(L)^ Rama mode was also observed in the Raman experiment^5^. No extra active Raman mode of the molecular *Cmca* phase is found in the calculations, indicating the splitting of the Raman peak observed in the experiment at 25 GPa is due to a new unknown phase.

**Figure 3:**
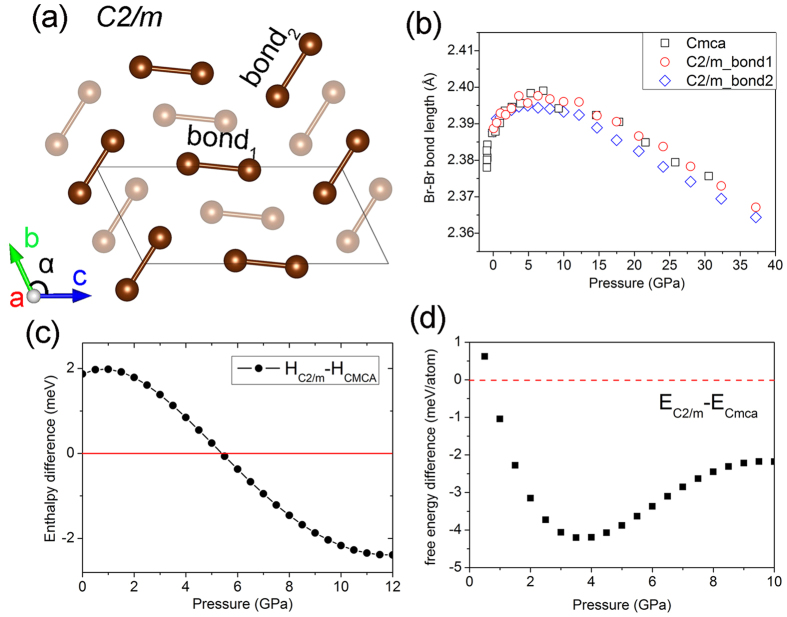
Results of the C2/m structure of bromine. (**a**) C2/m structure model. Bond_1_ and bond_2_ represent two different Br-Br intramolecular bonds in the structure. The faded atoms are in another layer. (**b**) Pressure evolution of the Br-Br bond length in the Cmca and C2/m phases. (**c**) Pressure dependent enthalpy differences between Cmca and C2/m phases of bromine. (**d**) Pressure dependent Gibbs free energy differences at 300 K between Cmca and C2/m phases.

In a study on solid iodine^7^, a new high pressure solid phase with space group *C*2/*m* was predicted to occur before the incommensurate phase V. The calculated Raman spectrum is in agreement with the splitting of the A_g_^(L)^ and B_3g_^(L)^ Raman bands at 12.5 GPa. Owing to the structural similarity between solid iodine and bromine, this new phase could be a candidate to explain the observed Raman splitting in solid bromine at 25 GPa. Figure 3a shows the Br_2_ *C*2/*m* structure optimized at 14.7 GPa. The relevant lattice parameters are *a* =  5.810 Å, *b* =  3.706 Å and *c* =  8.935 Å with α  =  114.492°. The two non-equivalent bromine atoms in the unit cell are located at (0, 0.9427, 0.1221) and (0.5, 0.1984, 0.3776). Similar to the iodine, there are two covalent intramolecular bonds in the *C*2/*m* structure of bromine. The calculated Br_2_ bond lengths of *Cmca* and *C*2/*m* phases under pressure are compared in Fig. 3b. The largest difference between bond_1_ and bond_2_ (illustrated in Fig. 3a) in the *C*2/*m* phase is only 0.005 Å at 24 GPa. This difference is much smaller than the magnitude of the bond length variation of ~0.035 Å from 0 to 40 GPa, and therefore cannot explain the anomalous bond length reversal.

**Figure 4:**
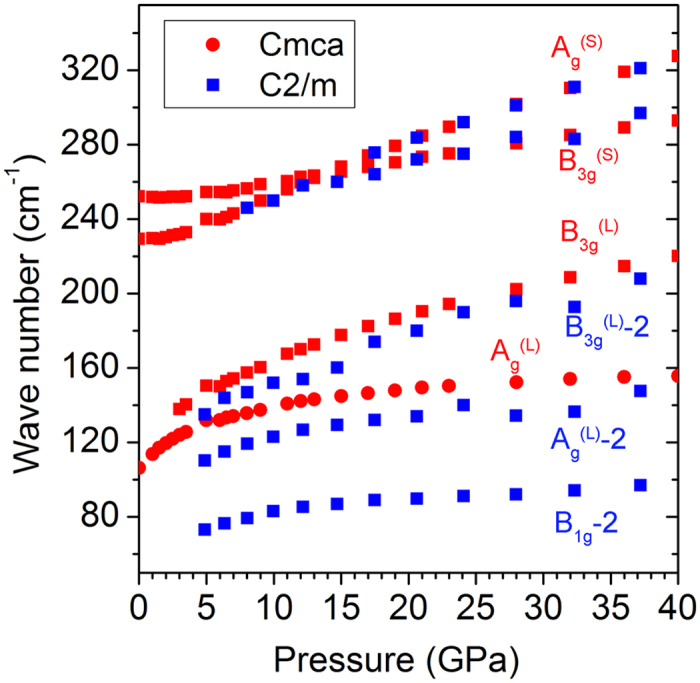
Pressure dependent vibrational frequencies of the Cmca phase (red square) and the C2/m phase (blue square).

The enthalpy (*H*) and Gibbs free energy (*E*_*Gibbs*_) at 300 K for the *Cmca* and *C*2/*m* structures of bromine are compared in Fig. 3(c,d). The Gibbs free energy including the vibrational entropy is given by,


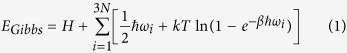


where *N* is the number of atoms in the cell, ω is the frequency of the phonon vibration mode, *k* is the Boltzman constant, *T* is the temperature, and *β* =  1/*kT*. The result clearly shows the *Cmca* and *C*2/*m* phases have very similar energies with the enthalpy and free energy differences less than 3 meV/atom and 5 meV/atom, respectively. However, the *C*2/*m* structure should become more stable at pressures over 5.5 GPa, at the pressure almost identical to the reversal of the Br-Br bond length! The theoretical results suggest *Cmca and C2/m* phases can coexist over a large pressure range. The calculated XRD spectra of these two phases at 14.7 GPa are compared in Fig. S5. The patterns are almost identical and this helps to explain that no noticeable structural change was observed from XRD measurements. Figure 4 shows the pressure dependence of the frequencies of active Raman modes in both *Cmca* and *C*2/*m* structure. The results show the splitting of A_g_^(L)^ and B_3g_^(L)^ bands of the *Cmca s*tructure and the emergence of a new lower frequency mode (B_1g_) at 25 GPa observed in the experiments can be assigned to the vibrational modes of the *C*2/*m* phase. Since there are almost no difference between the high frequency modes (A_g_^(S)^ and B_3g_^(S)^) between the two structures, leading to the apparent absence of Raman splitting of the high frequency vibrations. The main features in the observed Raman spectra have been reproduced. The present study explains the anomaly in the trend of the intramolecular bond length and changes in the Raman spectra. The results indicate that a new *C*2/*m* phase co-exist with the *Cmca* structure at 25 GPa before the onset of the incommensurate phase V.

## Conclusions

The structural evolution of the molecular solid phase of compressed bromine (*Cmca*) has been investigated with first-principles calculations. Although the absolute agreement between the calculated pressure with experiment is not satisfactory owing to the shortcomings in the description of weak intermolecular vdW interactions resulting in underestimated pressures, the anomalous bond length reversal and the observed splitting of the low frequency Raman bands are confirmed and explained. At low pressure the first neighbor intermolecular interaction plays a key role on enhancing the first neighbor interaction and thus weakens the intramolecular bond and elongated the Br-Br bonds. As pressure is increased further, the second neighbor intermolecular interaction becomes important and hinders the rotation of the bromine molecules. Consequently, further compression only shortened the bond length. The observed Raman splitting is shown to be a structural transformation to a new *C*2/*m* structure at the pressure where the Br-Br bond reached the maximum. The internal structures of the *Cmca* and *C*2/*m* phase are very similar and share the same trend in the Br-Br bond lengths as a function of pressure. The very similar free energies of both phases suggest possible co-existence over a broad pressure range. The present study not only reveals the origin of the pressure induced anomalous bond length change in the molecular bromine solid, but also predicted a new solid phase responsible for the previously observed but unexplained Raman splitting. The new findings encourage further experiments to confirm the structural variation and the new solid phase of bromine.

## Method

Electronic structure density functional theory (DFT) calculations were performed with plane wave basis set with projector augmented wave (PAW) potentials^14^ as implemented in the Vienna ab initio simulation package (VASP)^15^,^16^. Since bromine is a molecular solid at low pressure, the common density functionals are not expected to describe the weak interlayer and intermolecular vdW interactions correctly. A number of exchange-correlation (LDA^17^, PBE^18^, PBEsol^19^) and vdW functionals (DF^20^, DF2^21^, PBE-D2^22^, PBE-D3^23^, PBE-TS^24^, PBE-TS-SCS^25^) were tested. A plane wave energy cutoff of 600 eV was used. The Brillouin zone was sampled with a 12 ×  16 ×  8 *k*-point mesh. Test calculations show the total energies converged better than 1 meV/atom. In the geometry optimization calculation, the lattice vectors and the atomic coordinates were relaxed until the Hellmann–Feynman forces on all the atoms were less than 0.005 eV/Å.

## Additional Information

**How to cite this article**: Wu, M. *et al.* Anomalous bond length behavior and a new solid phase of bromine under pressure. *Sci. Rep.* **6**, 25649; doi: 10.1038/srep25649 (2016).

## Supplementary Material

Supplementary Information
