# Supplementary material for: Anomalous bond length behavior and a new solid phase of bromine under pressure
Source: Sci Rep. 2016 May 9;6:25649. doi: 10.1038/srep25649 (PMC4860644; doi:10.1038/srep25649)
Supplement: Supplementary Information [file srep25649-s1.pdf]

Supplementary Information

Anomalous bond length behavior and a new solid phase of bromine under pressure

Min Wu[1,2,3,*], John S. Tse[2,4,*], Yuanming Pan[3]

[1]College of Materials Science and Engineering, Zhejiang University of Technology, Hangzhou, 310014, P. R. China
[2]Department of Physics and Engineering Physics, University of Saskatchewan, Saskatoon, Saskatchewan, S7N 5E2 Canada
[3]Department of Geological Sciences, University of Saskatchewan, Saskatoon, Saskatchewan, S7N 5E2 Canada
[4]State Key Laboratory of Superhard Materials, Jilin University, Changchun 130012, P.R. China

Corresponding author: john.tse@usask.ca & wumindt2@163.com

Table. S1 Structural information of Br_2 in *Cmca* phase from calculations using different exchange-correlation and vdW functionals. r_1 *and* r_2 are the nearest interatomic distance and the second nearest interatomic distance, respectively. The unit of volume and distances are in $Å^3$ and Å. The experiment values are from ref. 9.

	**volume**	r_1	r_2	***a***	***b***	***c***
Exp.	260.570	2.270	3.310	6.670	4.480	8.720
LDA	219.600	2.402	2.930	6.641	3.930	8.415
PBE	317.780	2.394	3.129	8.565	4.266	8.697
PBEsol	246.620	2.397	2.989	7.196	4.017	8.533
DF	294.060	2.414	3.273	7.374	4.478	8.906
DF2	277.710	2.473	3.210	7.122	4.410	8.842
PBE-TS	264.590	2.391	3.099	7.268	4.243	8.580
PBE-TS-SCS	263.740	2.393	3.090	7.241	4.233	8.604
PBE-D2	245.120	2.387	3.108	6.738	4.217	8.628
PBE-D3	256.110	2.392	3.089	7.057	4.206	8.629

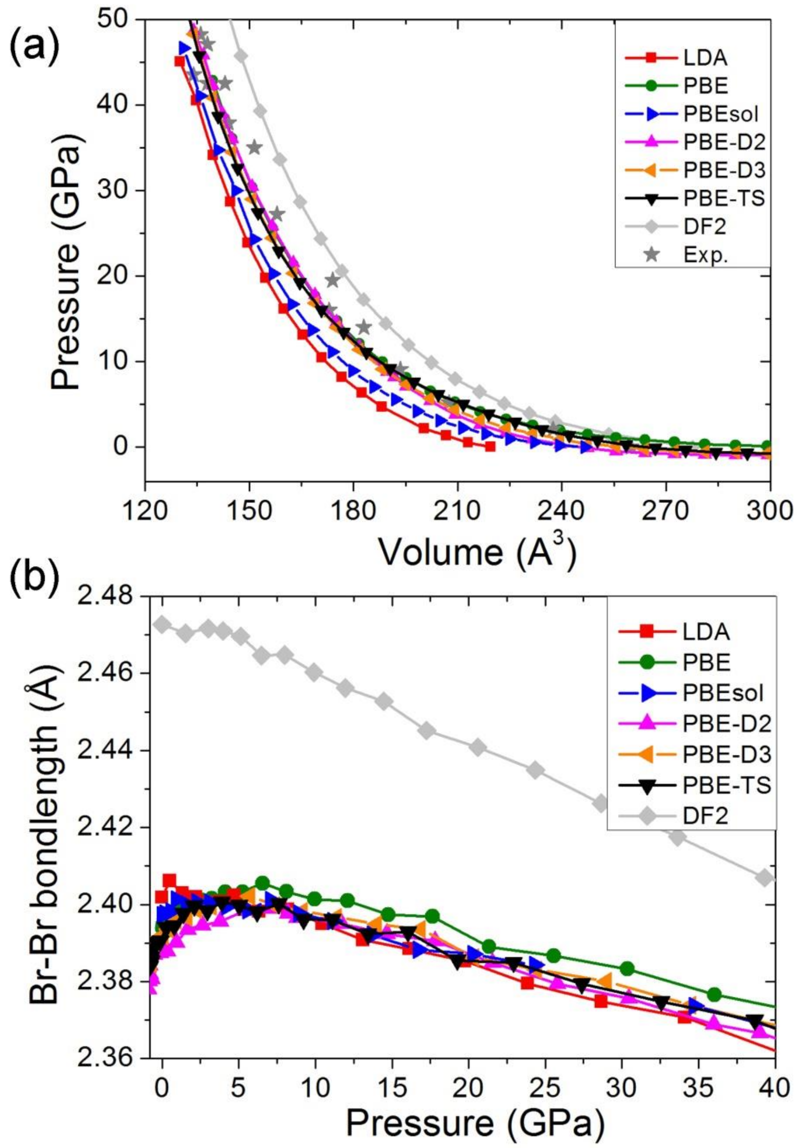

Fig. S1 (a) Equation of states of Bromine calculated using different density functionals. (b) Pressure dependent intramolecular bond length in the *Cmca* phase of bromine calculated by different methods.

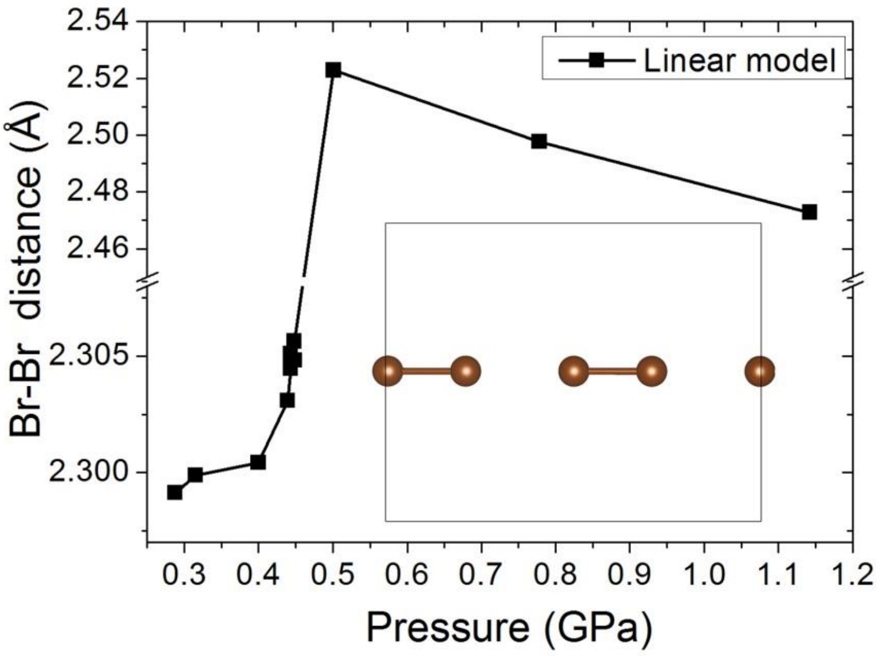

Fig. S2 The Br-Br bond length in the compressed 1D bromine model. The Br atoms in this 1D model are aligned in “head-to-toe” type in a straight line.

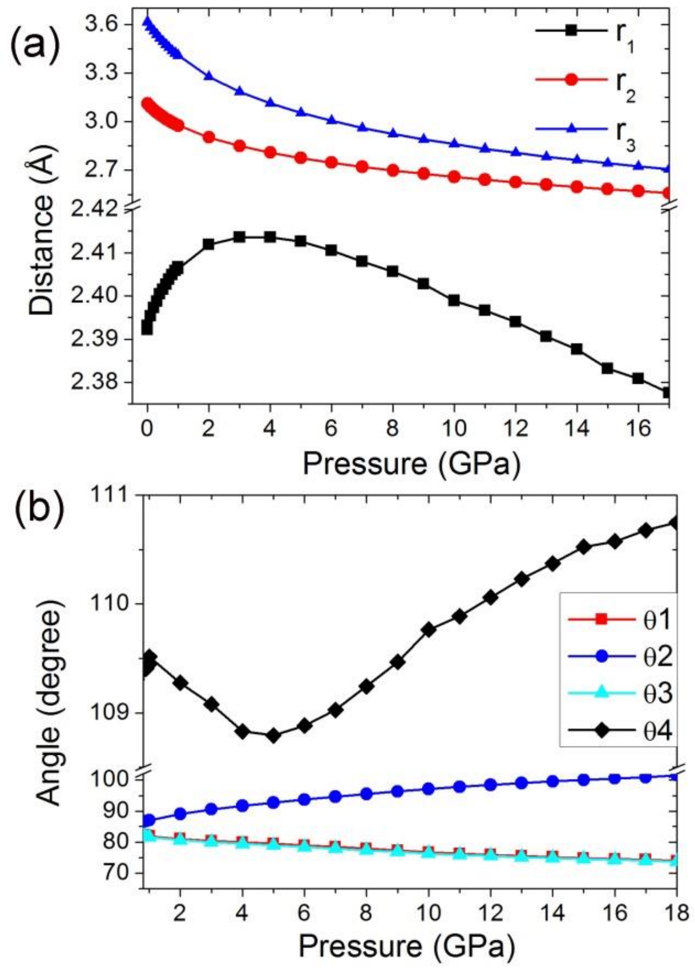

Fig. S3 (a) Interatomic distance under pressure in the bromine 2D model. (b) Intermolecular angles under pressure. During the geometry optimization calculations, the lattice vectors and the atomic coordination in the *bc* plane are fully optimized until the target pressure has been reached. The optimized structure is a perfect plane without any distortion along the direction perpendicular to the layer. The stress perpendicular to the *bc* plane is 0 in this 2D model.

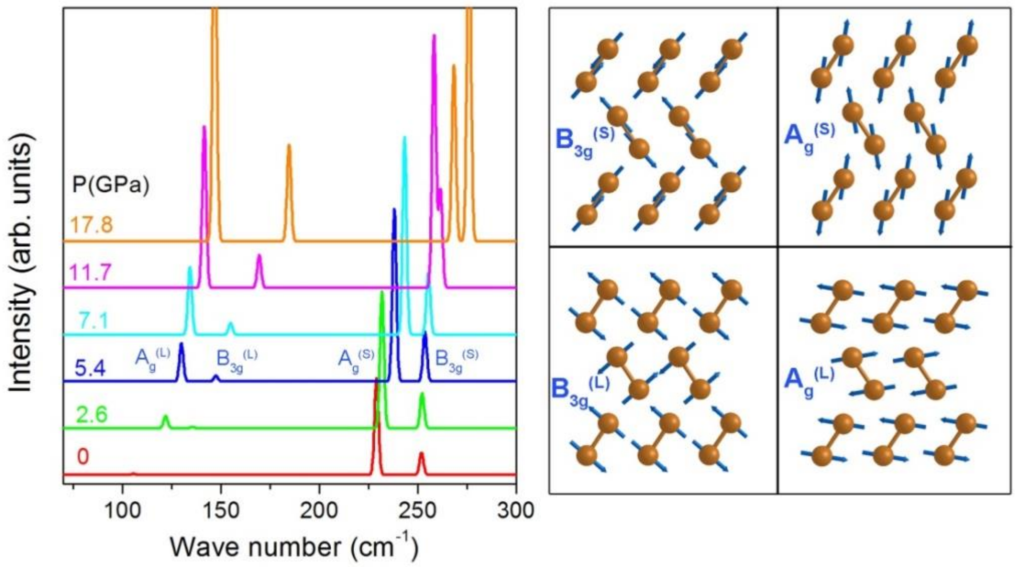

Fig. S4 Calculated Raman spectra of the bromine *Cmca* structure at selected pressures. The denoted four Raman modes are illustrated in the right panel.

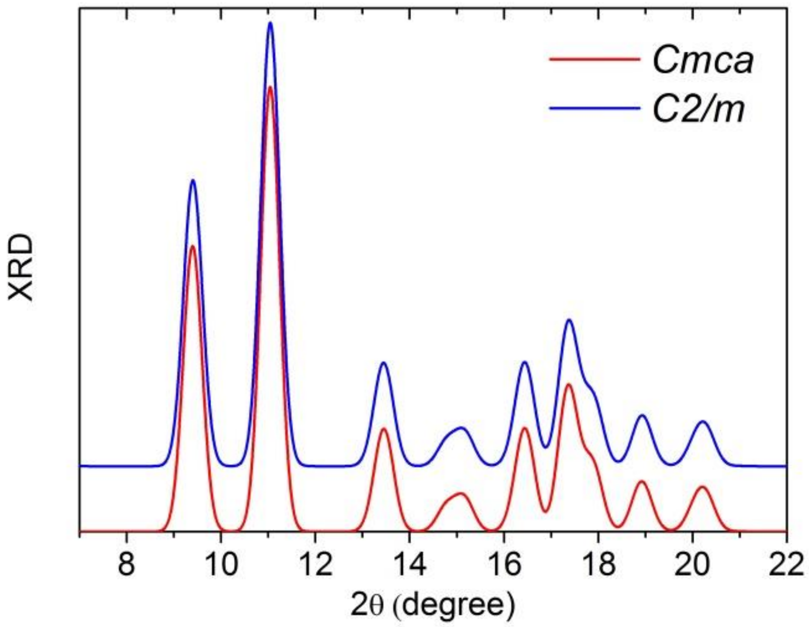

Fig. S5 Calculated X-ray diffraction patterns of the *Cmca* and *C2/m* bromine structures at 14.7 GPa (the X-ray wavelength λ is 0.48 Å).
